# Pressure-Induced Bandgap Engineering and Tuning Optical Responses of Cd_0.25_Zn_0.75_S Alloy for Optoelectronic and Photovoltaic Applications

**DOI:** 10.3390/ma15072617

**Published:** 2022-04-02

**Authors:** Muhammad Aamir Iqbal, Afaq Ahmad, Maria Malik, Jeong Ryeol Choi, Phuong V. Pham

**Affiliations:** 1 Centre of Excellence in Solid State Physics, University of the Punjab, Lahore 54590, Pakistan; aafaq.cssp@pu.edu.pk (A.A.); mariamalikc@gmail.com (M.M.); 2 Department of Nanoengineering, Kyonggi University, Suwon 16227, Korea; 3 Hangzhou Global Scientific and Technological Innovation Center, School of Micro-Nano Electronics, Zhejiang University, Hangzhou 310027, China

**Keywords:** bandgap engineering, Cd_0.25_Zn_0.75_S alloy, DFT, mBJ, pressure, tuned optical responses

## Abstract

The manipulation of composition and pressure, which affect the structure and, as a result, lead to new desired properties, is particularly significant for optimizing device performance. By considering the importance of pressure treatment, this study explores bandgap engineering and tuned optical responses of the ternary Cd_0.25_Zn_0.75_S alloy over a pressure range of 0–20 GPa using density functional theory. The functional material exhibits cubic symmetry at all pressures, and its bulk modulus increases with pressure. It is a direct bandgap semiconductor at Γ symmetry point, and its bandgap energy increases from 3.35 eV to 3.86 eV with an increase in pressure. Optical properties change with pressure, such that the absorption coefficient increases and absorbs near-ultraviolet light, while the static dielectric constant and static refractive index both increase with pressure. The effects of pressure on other optical parameters such as dielectric constant, extinction coefficient, refractive index, optical conductivity, and reflection are also explored. These findings provide significant theoretical guidance for the use of the Cd_0.25_Zn_0.75_S semiconductor in fabricating optoelectronic and photovoltaic devices functioning at varying pressure ranges and altitudes.

## 1. Introduction

The II-VI semiconductors of the type A_x_B_1−x_C (A = Cd, B = Zn, C = S, Se) are scientifically very important and their properties can be tuned for specific applications by adjusting the compositional factor along with pressure treatments for their use in well-known marketable optoelectronic devices that can work across entire spectral regions [1]. The optoelectronic industry is one of the key technological drivers of the economy, with the direct bandgap of CdZnS alloys finding their place in numerous appealing device applications owing to the remarkable growth in communication and information processing, display, and storage applications. [2]. These ternary alloys can be employed in the fabrication of light emitting diodes, lasers, variable wavelength photodetectors, light sensors, field emitters, solar cells, and photovoltaic devices [2,3].

Bandgap tailoring of II-VI direct and wide bandgap semiconductors has recently attracted substantial attention because of their potential applications in a variety of photonic and optoelectronic devices ranging from light emitters, solar cells, laser diodes, and electroluminescence [2,3,4,5,6]. The CdZnS ternary alloys are one of the wide bandgap semiconductors that have high stability and are used in the fabrication of photovoltaic, photoconductive, photoluminescent, photocatalytic, and luminescent devices [1,2,3,4,5,6,7]. Thin films of CdZnS alloys have been synthesized using limited solid solutions of cadmium–zinc sulfides [8], as well as a chemical bath deposition technique [9], whereas nanocrystals and nanocomposites have been reported using the successive ionic layer adsorption and reaction (SILAR) method [10], the sonochemical method [11], the hydrothermal method [12], the solvothermal method [13], the microemulsion technique [14], vapor transport chemical conversion [15], spray pyrolysis [16], and reverse pyrolysis [17]. Quantum dots of CdZnS alloys have also been prepared using sequential ionic layer adsorption and reaction techniques [18], the non-injection one-pot approach [19,20], and the co-precipitation method [21]. To examine the solar-to-hydrogen energy conversion efficiency and photocatalytic activity, porous Cd_1−x_Zn_x_S nanosheets with twinned phase junctions were manufactured using a cation-exchange method employing inorganic–organic hybrid ZnS diethylenetriamine nanoflakes with cadmium ions [22]. Photocurrent production in reduced graphene oxide–cadmium zinc sulfide nanocomposites under simulated solar light irradiation has been described for photodetection applications [23]. A structural, morphological, optical, and photocatalytic study was also conducted using plasmon-enhanced Au/Cd_1__−__x_Zn_x_S nanocomposites, and the Cd_0.25_Zn_0.75_S alloy bandgap was reported as 2.53 eV [24]. Theoretically, the thermodynamic properties of the ternary Cd_0.25_Zn_0.75_S alloy have been investigated using a quasi-harmonic model for pressures of 0–10 GPa and temperatures of 0–1200 K [25], whereas lattice thermal conductivity has been investigated for pressures of 0–10 GPa and temperatures of 300–1200 K, respectively [26]. Cd-concentration effects were also investigated on the structural, electronic, and optical properties of CdZnS alloys [27,28].

According to our understanding, the selected ternary Cd_0.25_Zn_0.75_S alloy has a significant deficiency in its behavior, and the lack of suitable information encouraged us to investigate its behavior under pressure. This research focuses on theoretical forecasting to provide analysis and further information on electronic and optical characteristics as well as to understand the underlying solid-state processes that occur when pressure is applied. The optical constants of a material evaluated under high pressure are very important in determining the material response and can be understood by studying the electronic band structures as the structure–property relationship is important in predicting the use of a material for possible device applications. Thermodynamically, pressure is a fundamental quantity that can be used to transfer matter from one state to another, therefore understanding the physical properties of materials under pressure can provide an early indication of how they will behave in real-world situations. The diamond anvil cell is an experimental setup suitable for performing luminescence and light-scattering experiments, especially at high pressures [29], and the steady development of this technique has opened up a vast field of high-pressure science. It is, therefore, appropriate that a reference database be provided to experimentalists and aim to improve the existing theoretical findings on this important class of materials. We have used the full-potential linearized augmented plane wave (FP-LAPW) method with functionals such as GGA, EV-GGA, and mBJ to forecast the electronic and optical properties in the framework of density functional theory.

In this study, we explored the pressure-induced bandgap engineering and optical responses of the ternary Cd_0.25_Zn_0.75_S alloy under varying hydrostatic pressures using DFT within the mBJ functional, and we then compared these findings to existing data at ambient pressure. This study is important because it is the first attempt to provide experimental reference data as well as being the first time that the band structures and optical responses of this ternary alloy have been examined at high pressures for possible photovoltaic and optoelectronic applications.

## 2. Theoretical Method

The self-consistent field full-potential linearized augmented plane wave (FP-LAPW) method, as implemented in the Wien2k code [30], is used to solve the Kohn–Sham equations using first-principles calculations based on density functional theory (DFT) [31]. This approach has become popular and essential for theoretical calculations, and its effectiveness has been shown by demonstrating the various results of its use. A supercell of ZnS was generated to incorporate Cd substitution with Zn atoms, in which 25% of Zn atoms are substituted with Cd atoms, and then optimized to find the lattice parameter to further investigate the physical properties, for which the core and valence states are separated by −6.0 Ry of energy and wave potentials in terms of spherical harmonics of the core are expanded up to L_max_ = 10. The convergence standard is set to be 0.00001 Ry, 0.00001 e and 1 mRy/Bohr for energy, charge, and force, respectively, where R_MT_ ∗ K_max_ = 7.0. The RMTs are set at 2.34, 2.34, and 1.92 Bohr for Cd, Zn, and S, respectively, while K-point sampling of a denser mesh 12 × 12 × 12 is generated in the full Brillouin zone. The band structures and optical responses under the influence of pressure are computed using the modified Becke–Johnson (mBJ) method, proposed by Tran and Blaha [32].

This study was mainly designed to explore band structures and optical responses under the influence of varying hydrostatic pressures and was accomplished by shaping the stability of this ternary alloy, for which structural parameters are important in materials physics as they allow the accumulation of information on the microscopic structure of materials and have a reasonably strong influence on the prediction of further characteristics. One can calculate the lattice constant of materials under the hydrostatic pressure effect using the relationship [33]; α(P) = α_0_[$1+P\frac{B\prime }{B_{0}}{]}^{\frac{-1}{3B^{\prime }}}$, where α_0_ is the equilibrium lattice constant, B_0_ is the bulk modulus, P is the hydrostatic pressure, and B′ is the pressure derivative of the bulk modulus, respectively. Many such theoretical studies have confirmed the success of this relationship in estimating the effect of pressure variations on the lattice constant and, as a result, on physical properties. The experimental results show that their respective outcomes are identical, which gives us confidence in using this relationship to investigate the effect of pressure on the electronic and optical properties of the ternary Cd_0.25_Zn_0.75_S semiconductor.

## 3. Results and Discussions

### 3.1. Electronic Properties

Electronic features, such as density of states (DOS) and band structures, are examined under rising pressure along the Brillouin zone with the highest symmetry points. The approximation of electronic features under varying pressures allows us to investigate and comprehend electronic behavior in order to forecast material utilization for specific device applications.

**Figure 1:**
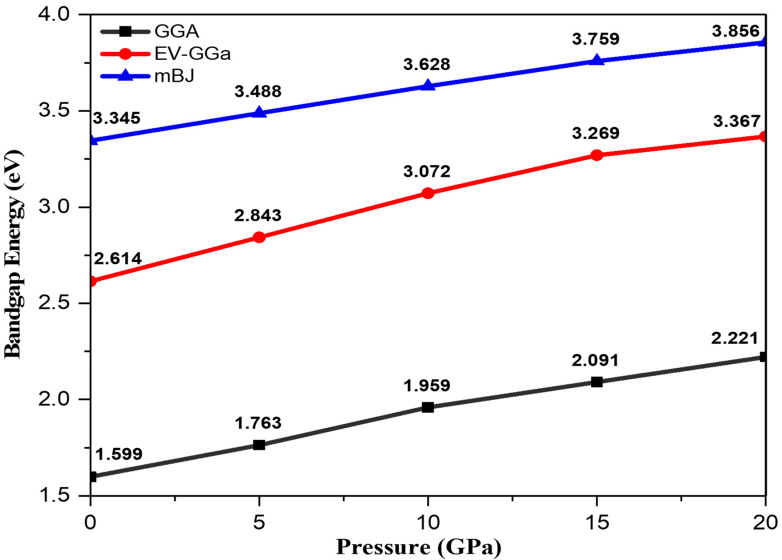
Bandgap variation as a function of pressure ranging from 0 to 20 GPa.

**Figure 2:**
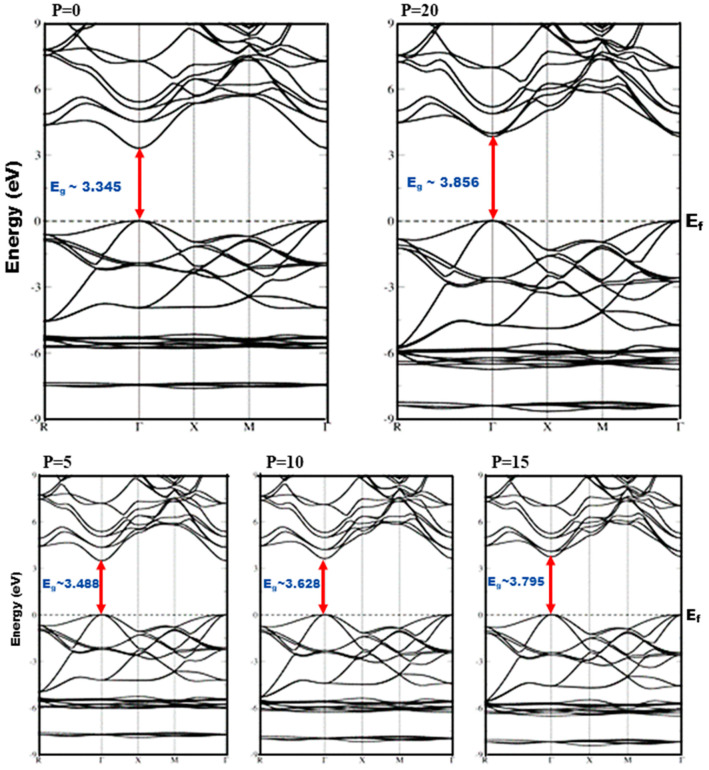
Band structures of Cd_0.25_Zn_0.75_S alloy computed within mBJ at pressures ranging from 0 to 20 GPa.

The pressure influence on band structures has been established by analyzing band structures in GGA, EV-GGA, and mBJ potentials at various pressures, whereas the pressure effect on the lattice constant is introduced by GGA and applied to band structures within EV-GGA and mBJ, respectively. GGA is widely recognized for underestimating the energy gap in self-consistent electronic band structure calculations using DFT [34], and to address this shortcoming, Engel and Vosko developed EV-GGA [35], a new functional formulation of GGA, which reproduces electronic bandgaps and yields better exchange potential in comparison to GGA. Therefore, we explored EV-GGA results along with GGA results and compared them to mBJ results; however, the results of these calculations indicate that the band structure’s overall features are identical, despite the difference in bandwidths. The band structure analysis demonstrates that this ternary alloy has a direct bandgap at all the considered pressures, and it is witnessed that the valence band maxima (VBM) and conduction band minima (CBM) are located at the same k-space point, thus this ternary alloy is a direct bandgap semiconductor, and the bandgap energy varies in the range of 3.35 eV to 3.86 eV within mBJ under the effect of pressure, as shown in Figure 1. The band structures calculated within different functionals (GGA, EV-GGA) are similar to mBJ; the only difference is in the band splitting value. As a result of the significant similarities between the band structures of EV-GGA and mBJ, the band structures of the mBJ functional are only shown in the cubic phase of crystal symmetry in Figure 2.

The substitution of 25% of Cd atoms with Zn creates impurity bands in the valence band, and the degeneracy of impurity bands increases with pressure enhancement. It is observed that the impurity bands are shifted towards lower energy values at high pressure, indicating an increase in bandgap energy. In addition, the valence band is more dispersed than the conduction band owing to the fact that they are more delocalized, and pressure has no significant influence on the conduction bandwidth but it does enhance the valence bandwidth. In general, if the valence bandwidth increases, the semiconductor material becomes less ionic, indicating a decrease in the ionic character of the Cd_0.25_Zn_0.75_S alloy under the influence of pressure. It should be noted that when pressure is applied, the maxima of the valence band shift downward while the minima of the conduction band shift upward, suggesting an increase in the bandgap with an increase in pressure. The GGA band structures at ambient pressure agree with those of 1.53 eV [27] and 1.60 eV [28], while the experimental results are 2.53 eV [24], 3.90 eV [36], and 4.25 eV [37]. This gives us the confidence to improve the bandgap using better functionals to overcome the underestimation of GGA results and further tune the optical responses to attain the desired device performance.

**Figure 3:**
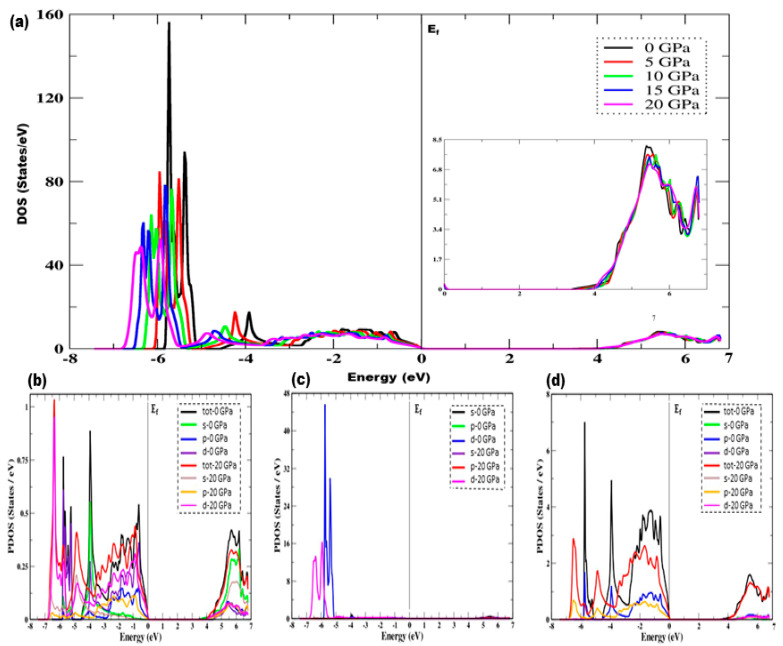
Density of states under the effect of pressure, (**a**) total density of states of Cd_0.25_Zn_0.75_S alloy, (**b**) partial density of states of Cd-atom, (**c**) partial density of states of S-atom, and (**d**) partial density of states of Zn-atom. The inset in (**a**) depicts a zoom of the conduction band around the Fermi level to display the variations in bandgap (right side) under the influence of pressure.

Total and partial density of states (DOS) can be used to present an in-depth understanding of band structures. From Figure 3, which depicts the density of states of the ternary Cd_0.25_Zn_0.75_S alloy under the effect of pressure, it can be witnessed that the pressure has a significant effect on the electron density of states of the valence band electrons, and the first peak that appears at −3.94 eV, corresponding to 0 GPa pressure, is shifted towards a lower energy value at −4.89 eV at 20 GPa pressure. The second peak that appears at −5.38 eV at 0 GPa is also shifted towards lower values at −5.93 eV at 20 GPa, with a significant decrease in peak height from 93.51 to 52.21 units under the influence of pressure. The main peak of the valence band, with a higher value of 155.44 units appearing at −5.77 eV, is also shifted towards the lower value of 48.09 units at 20 GPa under the effect of pressure. The peak intensity also decreases, and the highest peak intensity corresponds to ambient pressure. As the pressure effect starts to be considered, the peak height intensity starts to decrease with the enhancement in pressure range, while the overall trend of the density of states remains the same with the difference in peak height and a shift towards lower energies up to −6.81 eV in the valence band. Further, the electron density of states governed by the electrons of the conduction band does not have any significant effect under the influence of pressure. The inset in Figure 3a shows a zoom of the conduction band around the Fermi level to display the variation in bandgap under the influence of pressure, and it can be observed that the bandgap increases as the pressure is increased. Figure 3b–d depicts the partial density of states of Cd, S, and Zn atoms at 0 and 20 GPa pressures. It is clear that there is a significant change in the partial density of states of each atom, primarily in the valence band, where Cd-s, p, d states, and S-d states along with Zn-p states predominate, while the conduction band is primarily composed of Cd-s, p, and d states along with Zn-p and d states. The peak height is observed to be decreasing under the influence of pressure, with a shift towards lower energies.

### 3.2. Optical Properties

Radiation–matter interaction is of great importance in optoelectronics and photonics. When light interacts with matter, phenomena such as refraction, reflection, transmission, and absorption can be observed, and the response of matter is primarily determined by the frequency of the incident photon energy. The incident photon causes transition probabilities of an electron from an occupied state to an unoccupied state, resulting in a spectrum due to excitation, and this spectrum can be characterized using joint densities. The frequency-dependent complex dielectric function [38] can be used to investigate the system’s linear response.

(1) $$\varepsilon \left(\omega \right)=\varepsilon_{1}\left(\omega \right)+i\varepsilon_{2}\left(\omega \right)\;$$

where $\varepsilon_{1}\left(\omega \right)$ is the real and $\varepsilon_{2}\left(\omega \right)$ is the imaginary part of a complex dielectric function. The complex dielectric function yields useful information about how much material is polarized and can be characterized by static dielectric constant $\varepsilon_{1}\left(0\right)$, which represents the dielectric function at an irradiation frequency of zero. The real part, $\varepsilon_{1}\left(\omega \right)$, and the imaginary part, $\varepsilon_{2}\left(\omega \right)$, can be calculated using Kramers–Kronig transformations [39] as:

(2) $$\varepsilon_{1}\left(\omega \right)=1+\frac{2}{\pi }\int_{0}^{\infty }\frac{\omega^{/}\varepsilon_{2}\left(\omega \right)}{\omega^{/2}-\omega^{2}}d\omega^{/}\;$$

(3) $$\varepsilon_{2}\left(\omega \right)=-\frac{2\omega }{\pi }P\int_{0}^{\infty }\frac{\left(\varepsilon 1\left(\omega^{/}\right)-1\right)d\omega^{/}}{\omega^{/2}-\omega^{2}}\;$$

where ‘*P*’ and $\omega^{/}$ represent the momentum matrix and the joint density of states, respectively.

Optical constants such as the extinction coefficient and refractive index can be deduced from incident photon energy-dependent complex dielectric function. The refractive index, n(ω), provides useful information regarding the reduction in the speed of light when it enters a medium and depends on the frequency of the incident light [38]. The n(ω) and k(ω) can be approximated as:

(4) $$k\left(\omega \right)=\frac{1}{\sqrt{2}}{\left[\sqrt{\left\{{\varepsilon_{1}}^{2}\left(\omega \right)+{\varepsilon_{2}}^{2}\left(\omega \right)\right\}}-\varepsilon_{1}\left(\omega \right)\right]}^{1/2}$$

(5) $$n\left(\omega \right)=\frac{1}{\sqrt{2}}{\left[\sqrt{\left\{{\varepsilon_{1}}^{2}\left(\omega \right)+{\varepsilon_{2}}^{2}\left(\omega \right)\right\}}+\varepsilon_{1}\left(\omega \right)\right]}^{1/2}$$

Absorption of optical medium can be explained by the absorption coefficient [38], which is used to study the decay of light intensity per unit distance in material and can be deduced from the imaginary part k(ω) of the index of refraction as:

(6) $$\alpha \left(\omega \right)=\frac{4\pi k(\omega )}{\lambda_{0}}=\frac{\omega }{nc}\varepsilon_{2}\left(\omega \right)$$

The surface behavior of a material can be studied by observing the reflectance spectrum [38], which mathematically can be expressed as:

(7) $$R\left(\omega \right)=\frac{{\left(n\left(\omega \right)-1\right)}^{2}+k^{2}\left(\omega \right)}{{\left(n\left(\omega \right)+1\right)}^{2}+k^{2}\left(\omega \right)}\;$$

Optical conductance [38] is defined as the expansion of electrical transmission to high optical frequencies. It is a quantitative measurement that does not require any contact, is typically sensitive to charged responses, and can be deduced from the imaginary part of a complex dielectric function. The extinction coefficient k(ω) nearly follows $\varepsilon_{2}\left(\omega \right)$, and the optical conductivity can be determined by examining the variations in k(ω) governing with $\varepsilon_{2}\left(\omega \right)$. The real part of the optical conductance can be given as:

(8) Reσ(ω)=ω.ε24π 

The electron energy loss function [38] is given by:

(9) $$L\left(\omega \right)=lm\left(-\frac{1}{\varepsilon \left(\omega \right)}\right)=\frac{\varepsilon_{2}}{\left(\varepsilon_{1}^{2}\left(\omega \right)+\varepsilon_{2}^{2}\left(\omega \right)\right)}$$

**Figure 4:**
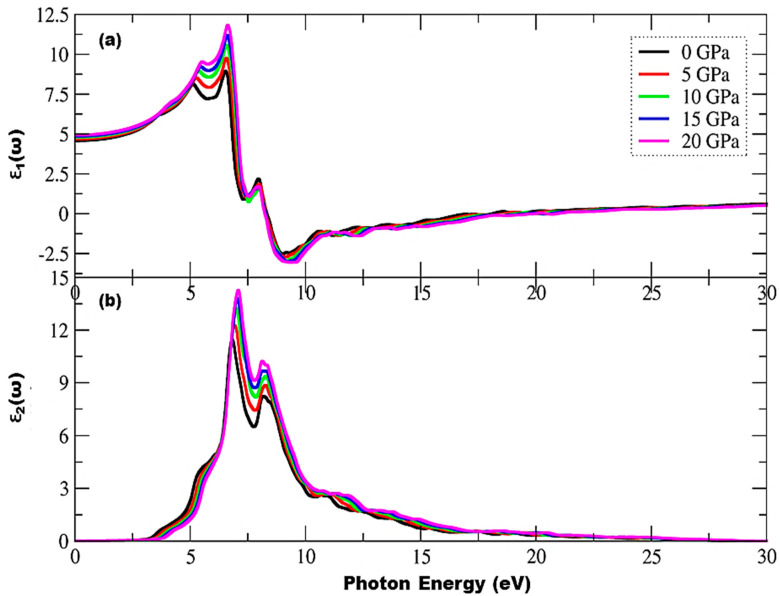
Pressure-induced variations in a complex dielectric function, (**a**) real part, and (**b**) imaginary part.

**Figure 5:**
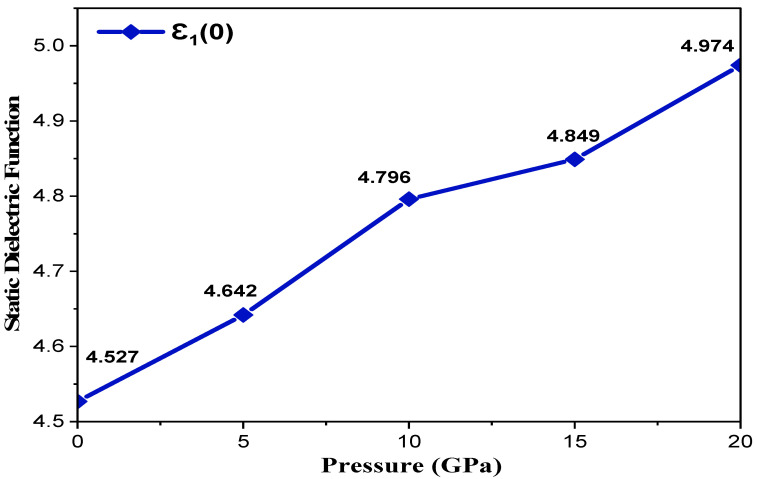
Pressure-induced variations in the static dielectric function.

**Figure 6:**
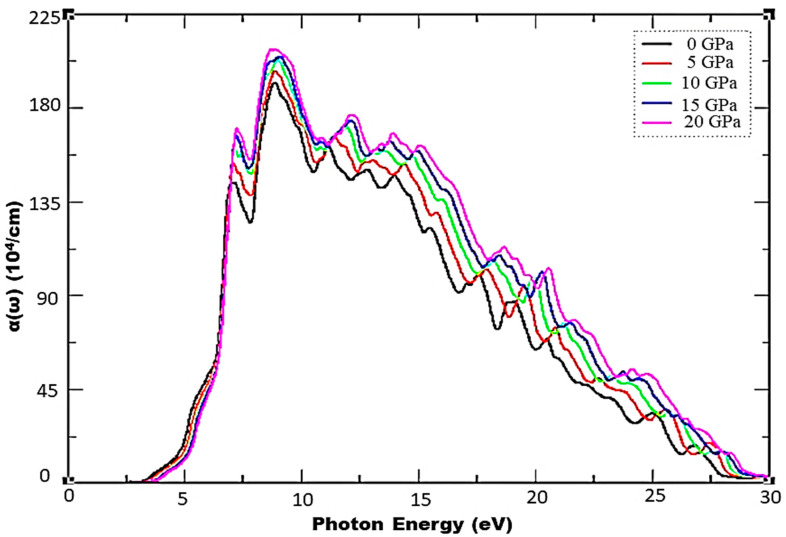
Pressure-induced variations in absorption.

There is a dependence of static dielectric function $\varepsilon_{1}$(0) on semiconductor bandgap ${Ε}_{g}$, and the Penn model [40] can be used to express their relationship as:

(10) $$\varepsilon_{1}\left(0\right)\approx 1+{\left(\frac{\hbar \omega_{p}}{{Ε}_{g}}\right)}^{2}\;$$

where plasma energy ($\hbar \omega_{p}$) is administered by plasma frequency ($\omega_{p}$). A denser mesh of k points was employed to investigate the optical characteristics of incident photon radiation up to 40 eV within mBJ for the Cd_0.25_Zn_0.75_S alloy with cubic symmetry and for isotropic optical response. Figure 4 depicts the complex dielectric function, and Figure 4a shows a trend of rapid increase in the real part of the complex dielectric function, followed by a small decrease, and then an increase up to the highest peak value of 8.91 units at 6.58 eV of incident radiation can be observed at 0 GPa, while 11.83 units at 6.63 eV can be observed at 20 GPa, respectively. It is also evident that $\varepsilon_{1}\left(\omega \right)$ has maximum transitions in the range of 3.56–7.98 eV, while peak height increases and shifts to higher $\varepsilon_{1}\left(\omega \right)$ values with an increase in pressure. This functional material exhibits a metallic nature below 0-unit values for incident radiation in the range of 8.25–17.98 eV as all radiation is reflected; hence, one can infer that this functional material may be utilized as a shield for a vacuum to extreme UV radiation. Negative $\varepsilon_{1}\left(\omega \right)$ becomes positive and almost static after 23.74 eV of incident radiation, showing that this functional material does not interact with high energy incident photons and hence can be used in optical lenses. The overall trend of dielectric function remains the same with the exception of an increase in peak height under the effect of pressure. The static dielectric constant, $\varepsilon_{1}\left(0\right)$, also increases with an increase in pressure, and its values are shown in Figure 5. As shown in Figure 4b, the threshold energies of $\varepsilon_{2}\left(\omega \right)\;$increase with pressure, and these critical points correspond to direct interband transitions from the highest valence band (Cd-s, d, Zn-p, and S-d states) to the lowest conduction band (Cd-s, d and Zn-p, d states). The threshold values start at 3.02 eV and the maximum peak value of 11.46 units corresponds to 6.81 eV of incident radiation at 0 GPa, while threshold values start at 3.74 eV and the maximum peak value of 14.26 units corresponds to 7.08 eV of incident radiation at 20 GPa, respectively, depicting an increase in threshold values under the influence of pressure. Beyond these critical points, there is a rapid increase of up to 7.08 eV of incident radiation. Two main peaks are observed in the region between the absorption threshold and 8.38 eV of incident photons. The lowest threshold value is observed at ambient pressure, and it increases under the pressure effect. Moreover, peak height increases from 11.46 to 14.26 units, which is shifted towards higher energies under the effect of pressure, and there is a static spectrum above 22.75 eV of incident radiation. Figure 6 shows the absorption spectrum, whereas $\varepsilon_{2}\left(\omega \right)$ has a deep effect on the absorption spectrum. Higher values of the imaginary part of the complex dielectric function correspond to larger values of the absorption coefficient. Understudy material shows an absorption increase of 191.65 to 208.54 units under the effect of pressure, while the maximum absorption peak corresponds to 20 GPa pressure at 8.68 eV of incident radiation. This material has high absorption above 7.25 eV of incident radiation, and it increases with an increase in pressure. A static trend is observed above 30 eV of incident photon energy, while no absorption spectrum is observed for photons having energies less than the bandgap energy. Higher absorption values are observed in the energy range of 7.04–12.09 eV and new peaks in the region 7.09–28.43 eV arise from electronic transitions of electrons in the valence band. Furthermore, a shift of peaks is observed towards higher values in the UV spectrum, and it can be seen from Figure 6 that the effect of pressure significantly enhances the absorptance and the peak height is also shifted towards higher values.

**Figure 7:**
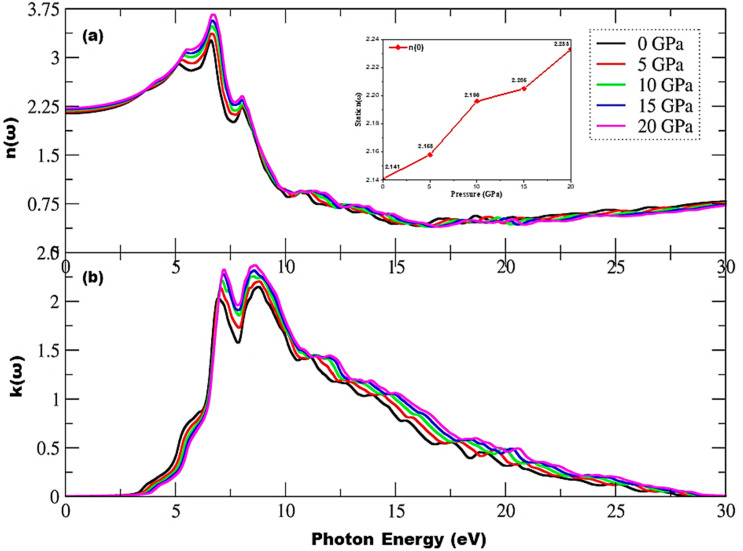
Pressure-induced variations in complex index of refraction, (**a**) refractive index, and (**b**) extinction coefficient. The inset in (**a**) depicts variations in static index of refraction, n(0), under the influence of pressure.

The optical properties of solids are frequency dependent, and as this material is cubic, the index of refraction is the same along the two transverse directions. Figure 7 depicts the index of refraction, n(ω), increasing with pressure alongside an increase in peak height from 3.25 to 3.68 units, and new peaks in the range of 3.53–8.04 eV of incident radiation are observed. As shown in the inset of Figure 7a, the pressure effect changes the static index of refraction, n(0), from 2.140 to 2.233 units, and the static response of this material can be seen in the spectrum at 30 eV of incident photon energy. The maximum peak height also increases with pressure as the extinction coefficient rapidly increases due to threshold energies, as shown in Figure 7b. The main peaks in the extinction coefficient range from 2.02 to 2.15 and from 2.32 to 2.37 at 0 and 20 GPa pressure, respectively, and no spectra are observed for k(ω) above 28.91 eV of radiation as this material does not interact with photons with energies greater than 28.91 eV, resulting in an ignorable absorptance spectrum. An overall increasing trend is observed in n(ω) and k(ω) under the pressure effect, with the active region corresponding to 3.16–15.09 eV of incident radiation.

**Figure 8:**
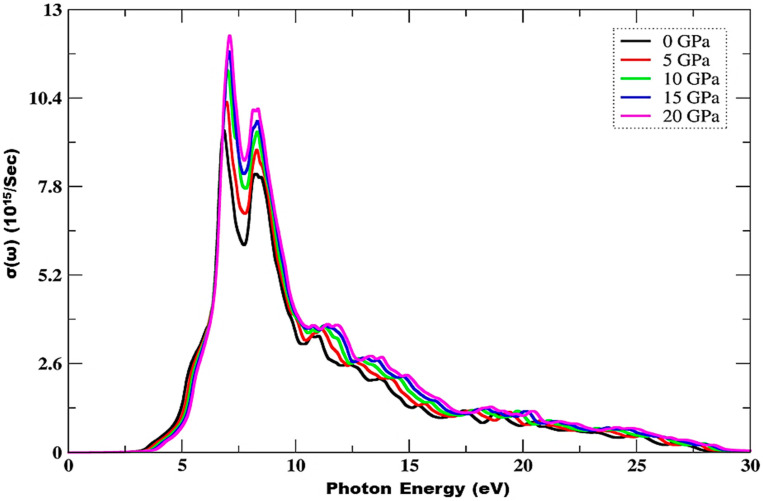
Pressure-influenced variations in optical conductance.

From Figure 8, it is clear that the optical conductance is zero as long as the incident photons have a lesser amount of energy than the bandgap energy. A rapid increase is observed from the spectrum, while peak height increases from 9.43 to 12.24 with an increase in pressure from 0 to 20 GPa. Maximum peaks are observed in the 6.81–8.52 eV range, and static response can be seen at 28.92 eV of incident radiation. At 20 GPa pressure, this material has a high optical conductance of 12.24 units, which corresponds to 7.12 eV of incident photon energy. It shows strong conductance in the range of 6.81–10.73 eV of incident radiation, which is shown to be mainly optically active in this region.

**Figure 9:**
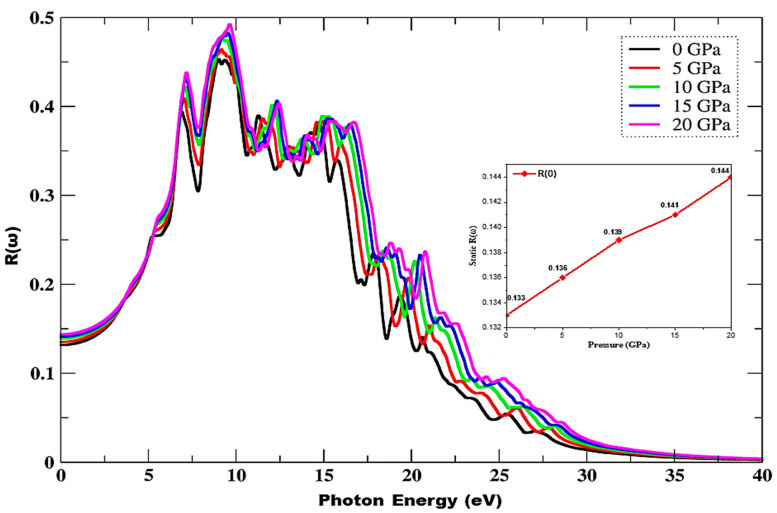
Pressure-influenced variations in reflectance. The inset depicts variation in static index of reflection, *R*(0), under the influence of pressure.

Figure 9 depicts a rapid increase in reflectance due to an increase in incident photon energy as well as in pressure and a shift of peaks towards higher values in combination with a wider spectrum, while peak height also increases under the influence of pressure. Dispersive reflectance humps are observed in the 11.26–26.94 eV range of incident radiation, and maximum reflectance is observed at 20 GPa, with 0.49 values at 9.59 eV. It can be witnessed from the inset of Figure 9 that the reflectance values increase as a function of pressure, and the static index of reflection is found to increase from 0.133 to 0.144 under the effect of pressure. New peaks are also observed in the 6.85–25.37 eV range of incident radiation.

**Figure 10:**
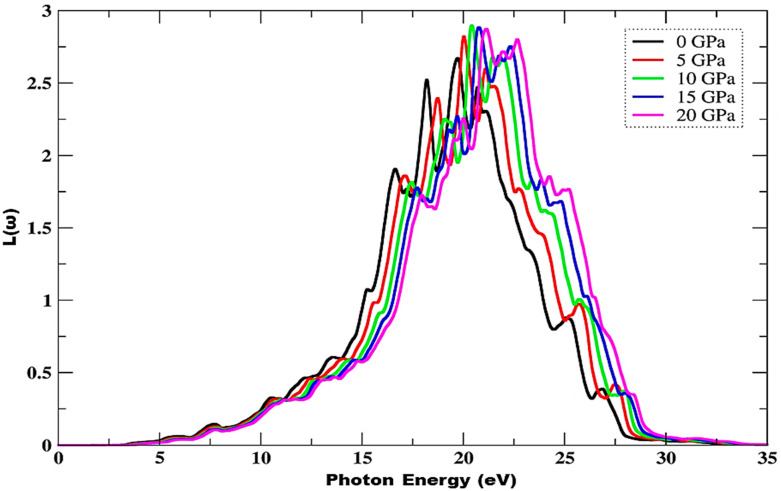
Pressure-influenced variations in energy loss function.

The energy loss function is zero below the bandgap energy of incident radiation, and it results in zero scattering of electrons. Above the bandgap energy, an inelastic scattering of electrons occurs, resulting in an energy loss spectrum that is directly proportional to the incident radiation. From Figure 10, it can be observed that the maximum energy loss function corresponds to a pressure of 10 GPa at 20.40 eV of incident radiation, having a value of 2.89 units, and it decreases with a further increase in pressure effect. Main peaks are observed in the 15.19–28.38 eV range, while the energy loss function is observed to be ignorable below threshold values and above 32.11 eV of incident photon energy. Moreover, the peak height is observed to be shifted towards higher L(ω) values under the effect of pressure.

## 4. Conclusions

This research explored the bandgap engineering and optical responses of a ternary Cd_0.25_Zn_0.75_S alloy under the effect of high pressure using DFT in which it is observed that this material exhibited cubic symmetry at all pressures. The computed band structures and density of states analyses revealed semiconductor properties, with a bandgap increase of 3.35 eV to 3.86 eV found with an increase in pressure component. Optical responses were also explored, and under the influence of pressure, the locations of all critical points were observed to shift towards higher energy. The peak trend was observed to remain the same, except for the peak height of the real and imaginary parts of the complex dielectric function, which increased. These electro-optic findings imply that this semiconductor can be used in the fabrication of optoelectronic and photovoltaic devices that work at a variety of pressures and altitudes.

## Data Availability

The data presented in this study are available on request from the corresponding author.
